# Chemokine CXCL13 mediates orofacial neuropathic pain via CXCR5/ERK pathway in the trigeminal ganglion of mice

**DOI:** 10.1186/s12974-016-0652-1

**Published:** 2016-07-11

**Authors:** Qian Zhang, De-Li Cao, Zhi-Jun Zhang, Bao-Chun Jiang, Yong-Jing Gao

**Affiliations:** Pain Research Laboratory, Institute of Nautical Medicine, Jiangsu Key Laboratory of Inflammation and Molecular Drug Target, Nantong University, Seyuan Road, Nantong, Jiangsu 226019 China; Department of Human Anatomy, School of Medicine, Nantong University, Nantong, Jiangsu 226001 China; Co-innovation Center of Neuroregeneration, Nantong University, Nantong, Jiangsu 226001 China

**Keywords:** CXCL13, CXCR5, Orofacial pain, Trigeminal ganglion, ERK, Proinflammatory cytokines

## Abstract

**Background:**

Trigeminal nerve damage-induced neuropathic pain is a severely debilitating chronic orofacial pain syndrome. Spinal chemokine CXCL13 and its receptor CXCR5 were recently demonstrated to play a pivotal role in the pathogenesis of spinal nerve ligation-induced neuropathic pain. Whether and how CXCL13/CXCR5 in the trigeminal ganglion (TG) mediates orofacial pain are unknown.

**Methods:**

The partial infraorbital nerve ligation (pIONL) was used to induce trigeminal neuropathic pain in mice. The expression of ATF3, CXCL13, CXCR5, and phosphorylated extracellular signal-regulated kinase (pERK) in the TG was detected by immunofluorescence staining and western blot. The effect of shRNA targeting on CXCL13 or CXCR5 on pain hypersensitivity was checked by behavioral testing.

**Results:**

pIONL induced persistent mechanical allodynia and increased the expression of ATF3, CXCL13, and CXCR5 in the TG. Inhibition of CXCL13 or CXCR5 by shRNA lentivirus attenuated pIONL-induced mechanical allodynia. Additionally, pIONL-induced neuropathic pain and the activation of ERK in the TG were reduced in *Cxcr5*^−/−^ mice. Furthermore, MEK inhibitor (PD98059) attenuated mechanical allodynia and reduced TNF-α and IL-1β upregulation induced by pIONL. TNF-α inhibitor (Etanercept) and IL-1β inhibitor (Diacerein) attenuated pIONL-induced orofacial pain. Finally, intra-TG injection of CXCL13 induced mechanical allodynia, increased the activation of ERK and the production of TNF-α and IL-1β in the TG of WT mice, but not in *Cxcr5*^−/−^ mice. Pretreatment with PD98059, Etanercept, or Diacerein partially blocked CXCL13-induced mechanical allodynia, and PD98059 also reduced CXCL13-induced TNF-α and IL-1β upregulation.

**Conclusions:**

CXCL13 and CXCR5 contribute to orofacial pain via ERK-mediated proinflammatory cytokines production. Targeting CXCL13/CXCR5/ERK/TNF-α and IL-1β pathway in the trigeminal ganglion may offer effective treatment for orofacial neuropathic pain.

## Background

Chronic neuropathic pain of orofacial region resulting from nerve trauma, compression, and/or demyelination is debilitating and often refractory to treatment. Neuroinflammation has been demonstrated to play an important role in the pathogenesis of neuropathic pain [1, 2]. A critical role in mechanisms of neuroinflammation is attributed to inflammatory mediators, including proinflammatory cytokines and chemokines, which augment the nociceptive signals at different anatomical locations, including peripheral nervous system and central nervous system (CNS) [1, 2]. Chemokines are a family of functionally related small secreted molecules with the established roles in the modulation of peripheral immune cell trafficking [3]. Increasing evidence has implicated spinal chemokines in chronic pain processing following nerve injury [4, 5]. However, little is known about chemokines in the trigeminal ganglion (TG) in mediating orofacial neuropathic pain.

C-X-C motif chemokine 13 (CXCL13), also known as B lymphocyte chemoattractant, was originally identified in stromal cells in B cell follicles to regulate homing of B cells and subsets of T cells [6, 7]. Previous studies showed that CXCL13 was not expressed in the healthy CNS but upregulated in the brain and spinal cord under some pathological conditions [8–10]. However, we recently found that CXCL13 was highly expressed in the lymph node and had low expression in the spinal cord of non-diseased humans [11]. CXCL13 was also expressed in the spinal cord of naïve mice and was upregulated after spinal nerve ligation (SNL) [11]. *Cxcl13* mRNA was also increased in the dorsal root ganglion (DRG) after DRG local inflammation or peripheral nerve injury [12].

The biological effects of chemokines are mediated via interaction with its G protein-coupled receptor (GPCR), and CXCR5 is the primary receptor of CXCL13. CXCR5 is expressed on all B cells and a subset of T cells in blood, lymphatic tissue, and cerebrospinal fluid [13, 14]. We recently found that CXCR5 was expressed in astrocytes in the spinal cord, and intrathecal injection of CXCL13 induced CXCR5-dependent pain hypersensitivity. Moreover, SNL-induced neuropathic pain was abrogated in *Cxcr5*^−/−^ mice [11], indicating the important role of CXCL13/CXCR5 signaling in the spinal cord in mediating neuropathic pain.

Mitogen-activated protein kinase (MAPK), including extracellular signal-regulated kinase 1/2 (ERK1/2), p38, and c-Jun *N*-terminal kinase (JNK) can be activated in the DRG and spinal cord by peripheral nerve injury [15]. Particularly, intrathecal injection of CXCL13 induced ERK activation in the spinal cord, and SNL-induced ERK activation was reduced in *Cxcr5*^−/−^ mice [11], suggesting that ERK is one of the downstream of CXCL13/CXCR5 signaling. Whether ERK can be activated by CXCL13/CXCR5 in the TG remains to be investigated.

The TG is a crucial site for pain transmission and pain modulation from the peripheral to the CNS in the oral maxillofacial region [16]. In the present study, we used partial infraorbital nerve ligation (pIONL) model to induce orofacial pain. We found that pIONL increased CXCL13 and CXCR5 expression in TG neurons, and CXCL13/CXCR5 was involved in orofacial mechanical allodynia. Our results also demonstrated that CXCL13/CXCR5 induces ERK activation and further augments neuroinflammation by increasing TNF-α and IL-1β in the TG.

## Methods

### Animals

Adult ICR mice (male, 8 weeks) were purchased from Experimental Animal Center of Nantong University. *Cxcr5*^−/−^ mice [B6.129S2 (Cg)-Cxcr5^tm1Lipp/J^, stock number 006659] were purchased from the Jackson Laboratory, and C57BL/6 wild-type mice were used as control. The animals were maintained on a 12:12 light–dark cycle at a room temperature of 22 ± 1 °C with free access to food and water. All animal procedures performed in this study were reviewed and approved by the Animal Care and Use Committee of Nantong University and were conducted in accordance with the guidelines of the International Association for the Study of Pain. Mice underwent a modified pIONL. In brief, the mouse was anesthetized with sodium pentobarbital and laid on the back. The oral cavity was exposed. A 1-mm longitudinal incision on the left buccal mucosa and at the level of the maxillary first molar was made to expose the infraorbital nerve (ION). The ION was then isolated and approximately one half of the nerve was tightly ligated with 8-0 silk suture and then transected just distal to the ligature. The buccal mucosa tissue was then sutured. The surgical procedure for the sham group was identical to that of the pIONL group, except that the ION was not ligated or transected.

### Behavioral testing

Animals were habituated to the testing environment daily for at least 2 days before baseline testing. All the behavioral experiments were done by individuals that were blinded to the treatment or genotypes of the mice.

#### Orofacial operant behavioral assessment

An Orofacial Stimulation Test (31300-002, Ugo Basile, Comerio VA, Italy) was used for the measurement of hypersensitivity to the mechanical stimulation of the trigeminal area [17, 18]. The apparatus consists of two parts: the plastic cage with the interface wall containing a drinking window, an infrared photo beam built on the exterior aspect of the window linked to the ORO software. The drinking window allows the mouse head to enter and acquire a reward (30 % milk), but the animal will be stimulated by a set of tungsten wire filaments attached around the drinking window. The animals initially underwent seven sessions of adaptation trainings in 1 a week without mechanical stimulator attached. For each training session, animals were first fasted for a 12-h period. Each mouse was then placed in a cage in which there was an Orofacial Stimulation Test System. During the test, the mouse was given 10 min to familiarize itself with its environment, and the drinking window was opened and the testing mouse was subsequently timed for 10 min to allow drinking the milk. The contact number and total duration of time the mouse spent acquiring the reward were recorded and analyzed with the ORO software.

#### Von Frey test

For experiments that several time points are needed to be checked in 24 h, Von Frey test was used, as orofacial stimulation test can be checked only once a day. For the Von Frey test, the mice were put in metal mesh boxes and allowed 30 min for habituation before examination. A graded series of Von Frey filaments was used for mechanical stimulation of the ipsilateral infraorbital nerve territory (0.02–2.56 g, Stoelting, Wood Dale, IL). The threshold was taken with a response of a brisk withdrawal of the head. The 50 % head withdrawal threshold was determined using Dixon’s up-down method [19].

### Drugs and administration

Recombinant murine CXCL13 was purchased from PeproTech (Rocky Hills, NJ). MEK inhibitor PD98059 was purchased from Merck KGaA (Darmstadt, Germany). TNF-α inhibitor (Etanercept) and IL-1β inhibitor (Diacerein) were purchased from Pfizer (New York, NY) and Selleck Chemicals (Westlake Village, CA), respectively. The drugs were injected with a 30 G needle from the infraorbital foramen to the foramen rotundum. The tip of the needle terminated at the medial part of the trigeminal ganglion, and the reagents (5 μl) was slowly delivered to the trigeminal ganglion [20].

### Real-time quantitative PCR (qPCR)

The total RNA of the TG was extracted using Trizol reagent (Invitrogen). One microgram of total RNA was reverse transcribed using an oligo(dT) primer according to the manufacturer’s protocol (Takara, Shiga, Japan). qPCR analysis was performed in the Real-time Detection System (Rotor-Gene 6000, Hamburg, Germany) by SYBR green I dye detection (Takara). The following primers were used: *Cxcl13* forward, 5′-GGC CAC GGT ATT CTG GAA GC-3′; *Cxcl13* reverse, 5′-ACC GAC AAC AGT TGA AAT CAC TC-3′; *Cxcr5* forward, 5′-TGG CCT TCTA CAG TAA CAG CA-3′; *Cxcr5* reverse, 5′-GCA TGA ATA CCG CCT TAA AGG AC-3′; *Tnf-α* forward, 5′-GTT CTA TGG CCC AGA CCC TCA C-3′; *Tnf-α* reverse, 5′-GGC ACC ACT AGT TGG TTG TCT TTG-3′; *Il-1β* forward, 5′-TCC AGG ATG AGG ACA TGA GCA C-3′; *Il-1β* reverse 5′-GAA CGT CAC ACA CCA GCA GGT TA-3′; *Gapdh* forward, 5′-GCT TGA AGG TGT TGC CCT CAG-3′; *Gapdh* reverse, 5′-AGA AGC CAG CGT TCA CCA GAC-3′. The PCR amplifications were performed at 95 °C for 30 s, followed by 40 cycles of thermal cycling at 95 °C for 5 s and 60 °C for 45 s. *Gapdh* was used as endogenous control to normalize differences. Melt curves were performed on completion of the cycles to ensure that nonspecific products were absent. Quantification was performed by normalizing Ct (cycle threshold) values with *Gapdh* Ct and analyzed with the 2-^ΔΔCT^ method.

### Western blot

Animals were transcardially perfused with PBS. The ipsilateral trigeminal ganglia were dissected and homogenized in a lysis buffer containing protease and phosphatase inhibitors (Sigma, St Louis, MO). Protein concentrations were determined by BCA Protein Assay (Pierce, Rockford, IL). Protein samples (30 μg) were separated on SDS–PAGE gel and transferred to nitrocellulose blots. The blots were blocked with 5 % milk and incubated overnight at 4 °C with antibody against CXCL13 (Goat, 1:100, Santa Cruz, Dallas, Texas), CXCR5 (rabbit, 1:100, Santa Cruz), pERK (rabbit, 1:500, Cell Signaling, Beverly, MA), ERK (rabbit, 1:500, Cell Signaling), and GAPDH (mouse, 1:20000, Millipore, Billerica, MA). These blots were further incubated with IRDye 800CW secondary antibodies for 2 h at room temperature and captured by Odyssey Imaging System (LI-COR Bioscience, Lincoln, NE). Specific bands were evaluated by apparent molecular size. The intensity of the selected bands was analyzed using Image J software (NIH, Bethesda, MD).

### Immunohistochemistry

Animals were deeply anesthetized with isoflurane and perfused through the ascending aorta with PBS followed by 4 % paraformaldehyde in 0.1M PB. After the perfusion, the ipsilateral TG was removed, postfixed, and cryo-protected by 20 % sucrose. TG sections (14 μm) were cut in a cryostat and processed for immunofluorescence as we described previously [21]. The sections were first blocked with 8 % goat or donkey serum for 2 h at room temperature, then incubated overnight at 4 °C with the following primary antibodies: ATF3 (Rabbit, 1:1000, Santa Cruz), CXCL13 (goat, 1:100, Santa Cruz), CXCR5 (rabbit, 1:100; Santa Cruz), neuronal specific marker β-III tubulin (Mouse, 1:500, R&D), and pERK (rabbit, 1:500, Millipore). The sections were then incubated for 2 h at room temperature with Cy3-conjugated secondary antibodies or Alexa 488-conjugated secondary antibodies (1:1000, Jackson ImmunoResearch, West Grove, PA). The stained sections were examined with a Leica fluorescence microscope, and images were captured with a CCD Spot camera. The specificity of the CXCL13 antibody and CXCR5 antibody were checked by absorption experiment or in *Cxcr5* KO mice, respectively [11].

### Lentiviral vectors production and intra-TG injection

The shRNA targeting the sequence of mice *Cxcl13* (Gene Bank Accession: NM_018866) or *Cxcr5* (Gene Bank Accession: NM_007551) was designed respectively. An additional scrambled sequence was also designed as a negative control (NC). The recombinant lentivirus containing *Cxcl13* shRNA (LV-*Cxcl13* shRNA), *Cxcr5* shRNA (LV-*Cxcr5* shRNA), or NC shRNA (LV-NC) was packaged using pGCSIL-GFP vector by GeneChem (Shanghai, China). The sequences of the shRNAs are: *Cxcl13* shRNA, 5′-TCG TGC CAA ATG GTT ACA A-3′; *Cxcr5* shRNA, 5′-CCA TCA CCT TGT GTG AAT T-3′; NC shRNA, and 5′-TTC TCC GAA CGT GTC ACG T-3′. The lentivirus (8 × 10^5^ TU, 1 μl) was injected into the trigeminal ganglion through the infraorbital foramen using a 30 G needle.

### Quantification and statistics

All data were expressed as mean ± SEM. The behavioral data were analyzed by two-way repeated measures ANOVA followed by Bonferroni test as the post hoc multiple comparison analysis. For Western blot, the density of specific bands was measured with ImageJ. CXCL13 and CXCR5 levels were normalized to GAPDH, and pERK levels were normalized to total ERK [22]. Differences between groups were compared using one-way ANOVA followed by Bonferroni test or using Student’s *t* test if only two groups were applied. The criterion for statistical significance was *P* < 0.05.

## Results

### pIONL induces persistent mechanical allodynia and ATF3 expression in the TG

**Fig. 1:**
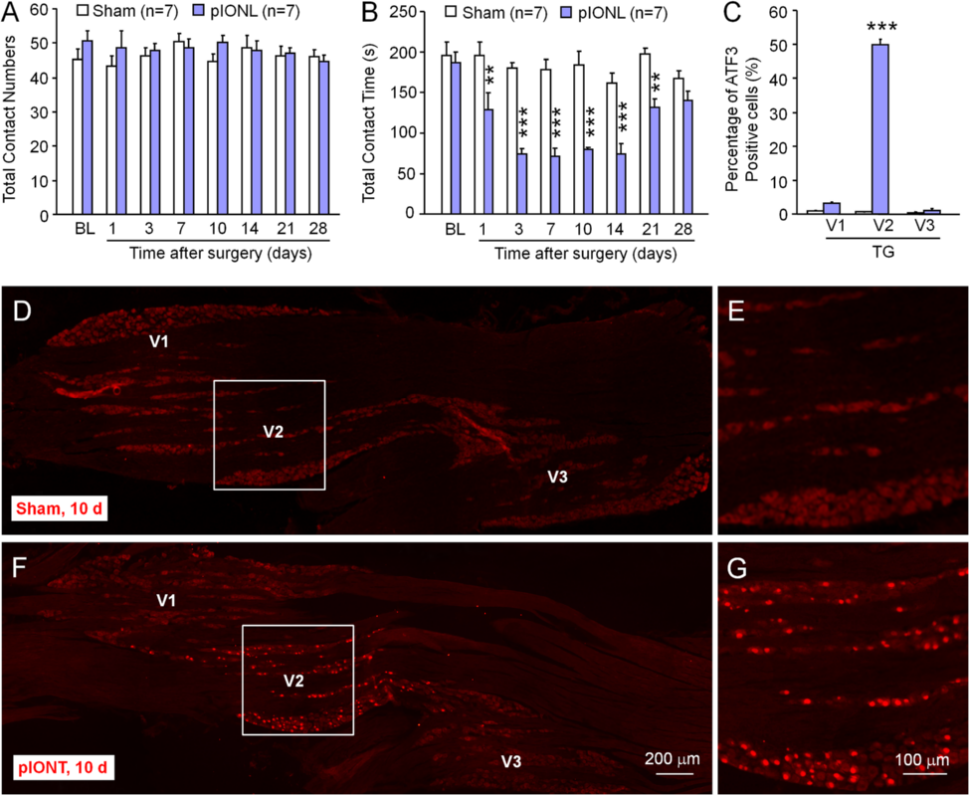
pIONL induces mechanical allodynia assessed by orofacial operant test and increases ATF3 expression in the maxillary division of TG neurons. **a** Total contact number of orofacial operant test with mechanical stimulation was comparable between sham and pIONL mice. *P* > 0.05. Two-way repeated measures ANOVA. **b** Total contact time was significantly decreased from 1 to 21 days after pIONL, compared to sham group. **P < 0.01, ***P < 0.001, pIONL vs. sham. Two-way repeated measures ANOVA followed by Bonferroni test. **c** The percentage of ATF3-positive cells were dramatically increased in the maxillary division (V2) of TG 10 days after pIONL, whereas ATF3-positive cells were not significantly changed in the ophthalmic (V1) division or mandibular (V3) division. ***P < 0.001, pIONL vs. sham. Student’s *t* test. **d**–**g** Representative images show the expression of ATF3 in the TG of sham-treated (**d, e**) or pIONL (**f**, **g**) animals. **e, g** High-magnification images of **d** and **f**, indicated in the *white boxes*

Before and after pIONL or sham operation, we performed orofacial operant tests to check mechanical allodynia. Both total contact number and contact time were recorded and analyzed. As shown in Fig. 1a, total contact number was not significantly changed after pIONL, and no significant difference was found between sham and pIONL groups (*P* > 0.05, two-way repeated measures ANOVA). However, total contact time in pIONL group was reduced 1 day after the operation, maintained from day 3 to day 14, began to recover toward baseline at day 21 and fully recovered at day 28 (*P* < 0.001, two-way repeated measures ANOVA), whereas the sham-operated animals showed similar contact time during all the time points (*P* > 0.05, two-way repeated measures ANOVA, Fig. 1b). These data indicate that the reduction of total contact time in pIONL group is not due to the decrease of animals’ attempt to drink milk [23]. In addition, the time course of pIONL-induced mechanical allodynia reflected by the reduction of contact time is comparable to the test by Von Frey filaments [24], suggesting that orofacial operant test is reliable, and pIONL induces persistent mechanical allodynia.

**Fig. 2:**
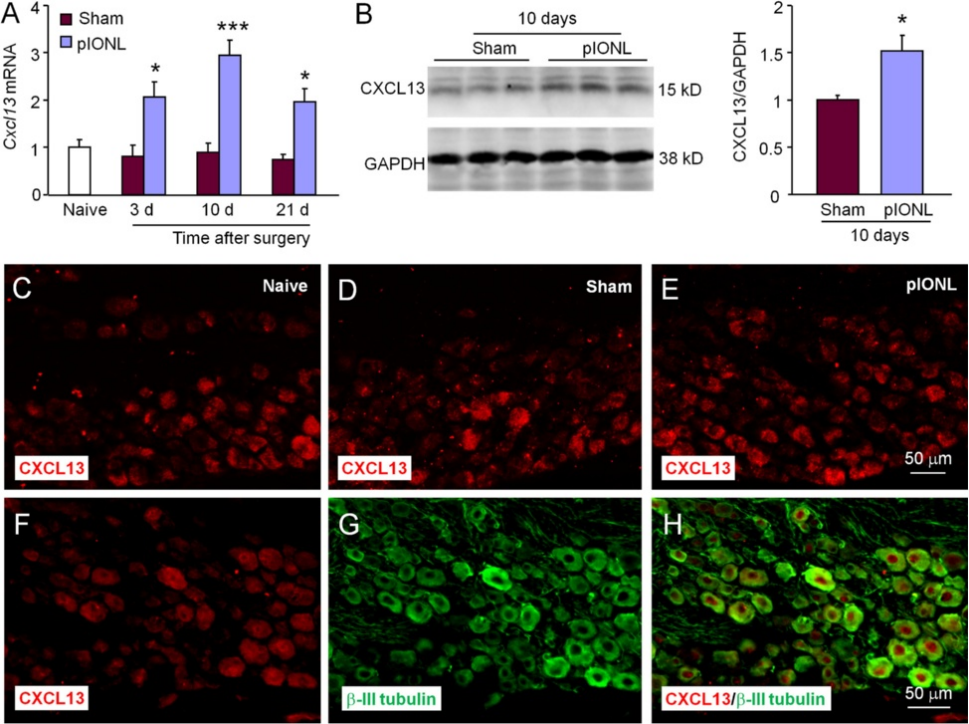
pIONL induces persistent CXCL13 expression in the TG. **a** The time course of *Cxcl13* mRNA expression in the ipsilateral TG from naïve, sham, and pIONL-operated mice. pIONL increased *Cxcl13* expression at 3, 10, and 21 days, compared to sham. *P < 0.05, ***P < 0.001. Two-way ANOVA followed by Bonferroni test. **b** Western blot shows increased CXCL13 protein level 10 days after pIONL, compared to sham. *P < 0.05. Student’s *t* test. **c** Representative images of CXCL13 immunofluorescence in the TG. CXCL13-IR was low in naïve mice (**c**) and sham mice (**d**), but increased in the TG of pIONL mice (**e**). **f**–**h** Double staining of CXCL13 (**f**) and neuronal marker β-III tubulin (**g**) shows the neuronal expression of CXCL13

We then checked the expression of ATF3, a marker for nerve injury in the ipsilateral TG 10 days after operation. ATF3-immunoreactivity (IR) was not shown in the TG of sham-operated mice (Fig. 1c–e). However, pIONL dramatically increased the percentage of ATF3-IR neurons in the maxillary (V2) division of TG from 0.5 ± 0.1 to 49.8 ± 0.7 % (*P* < 0.001, Fig. 2c, f, g). The percentage of ATF3-IR cells was not significantly changed in the ophthalmic (V1) division or mandibular (V3) division (*P* > 0.05, pIONL vs. sham, Fig. 1c). These data confirmed the injury of the second branch of the trigeminal nerve by pIONL.

### pIONL induces persistent CXCL13 upregulation in the TG

We then checked the expression of *Cxcl13* in the ipsilateral TG after pIONL or sham-operation. *Cxcl13* mRNA was significantly increased at days 1, 10, and 21 in pIONL mice compared to sham-operated mice (*P* < 0.05 or 0.001, pIONL vs. sham, two-way ANOVA followed by Bonferroni test, Fig. 2a). The mRNA level did not significantly differ between naïve and sham-operated mice at all the time points (*P* > 0.05, one-way ANOVA, Fig. 2a). We further checked CXCL13 protein level by western blot. As shown in Fig. 2b, pIONL significantly increased CXCL13 protein compared to sham operation (*P* < 0.05, Student’s *t* test). Immunostaining further showed that CXCL13 was expressed in the TG of naïve animals (Fig. 2c) and sham-operated animals (Fig. 2d). pIONL markedly increased CXCL13 expression 10 days after operation (Fig. 2e). Double staining of CXCL13 with neuronal marker β-III tubulin showed that CXCL13 was expressed in neurons (Fig. 2f–h). These data suggest the possible involvement of CXCL13 in pIONL-induced orofacial pain.

### Inhibition of CXCL13 by shRNA lentivirus alleviates pIONL-induced mechanical allodynia

**Fig. 3:**
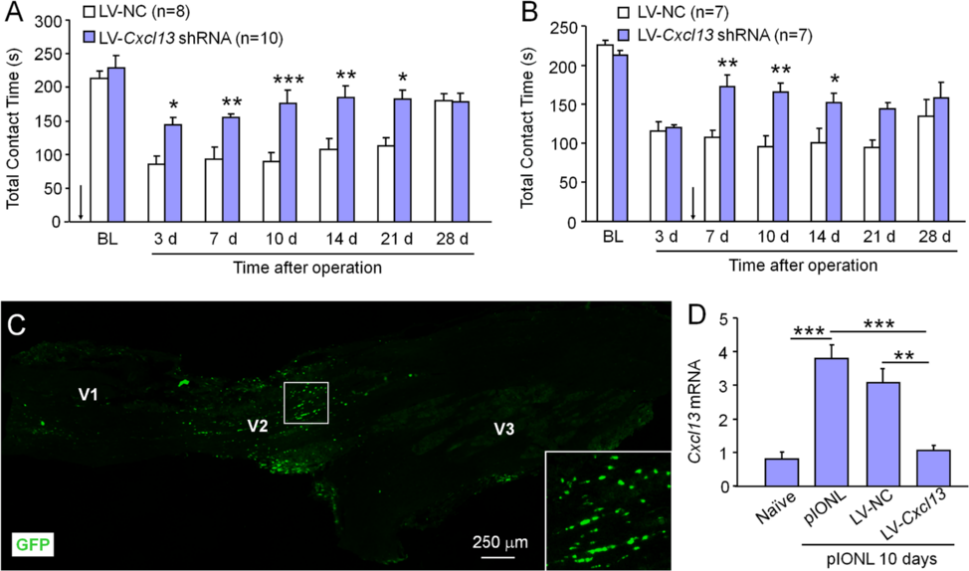
Inhibition of CXCL13 by shRNA lentivirus attenuated pIONL-induced mechanical allodynia. **a** Pretreatment with LV-*Cxcl13* shRNA increased the contact time between 3 and 21 days after the operation, compared to LV-NC-injected mice. Intra-TG injection was performed 7 days before pIONL (*arrow*). *P < 0.05, **P < 0.01, ***P < 0.001. Two-way repeated measures ANOVA followed by Bonferroni test. **b** Posttreatment with LV-*Cxcl13* shRNA increased the contact time between 7 and 14 days after the operation, compared to LV-NC treatment. Intra-TG injection was performed 3 days after pIONL (*arrow*). *P < 0.05, **P < 0.01. Two-way repeated measures ANOVA followed by Bonferroni test. **c** Representative fluorescence photomicrograph shows GFP expression in the TG 7 days after intra-TG infusion of lentivirus vector. **d** Real-time PCR assay of *Cxcl13* shows that pretreatment with LV-*Cxcl13* shRNA inhibited pIONL-induced *Cxcl13* upregulation. **P < 0.01, ***P < 0.001. Student’s *t* test

To examine the role of CXCL13 in the development and maintenance of trigeminal neuropathic pain, we injected *Cxcl13* shRNA lentivirus vectors (LV-*Cxcl13* shRNA) into the TG before or after pIONL. Our previous study has shown that LV-*Cxcl13* shRNA effectively decreased CXCL13 expression both in vitro and in vivo [11]. As shown in Fig. 3a, compared to control lentivirus injection (LV-NC), pretreatment with LV-*Cxcl13* shRNA (7 days before pIONL) inhibited pIONL-induced mechanical allodynia for more than 21 days (*P* < 0.001, two-way repeated measures ANOVA). In addition, posttreatment with LV-*Cxcl13* shRNA (3 days after pIONL) also attenuated mechanical allodynia from day 7 to day 14 (*P* < 0.001, two-way repeated measures ANOVA, Fig. 3b). These data suggest that CXCL13 plays an important role in both development and maintenance of trigeminal neuropathic pain.

To confirm the knockdown effect of LV-*Cxcl13* shRNA, we checked green fluorescent protein (GFP) expression 7 days after the injection and found GFP fluorescence in the maxillary division of TG (Fig. 3c). In addition, pretreatment of LV-*Cxcl13* shRNA inhibited pIONL-induced *Cxcl13* mRNA increase 10 days after pIONL (*P* < 0.01, vs. LV-NC), whereas LV-NC had no effect on *Cxcl13* expression (*P* > 0.05, vs. pIONL, Fig. 3d).

### pIONL induces persistent CXCR5 upregulation in the TG

**Fig. 4:**
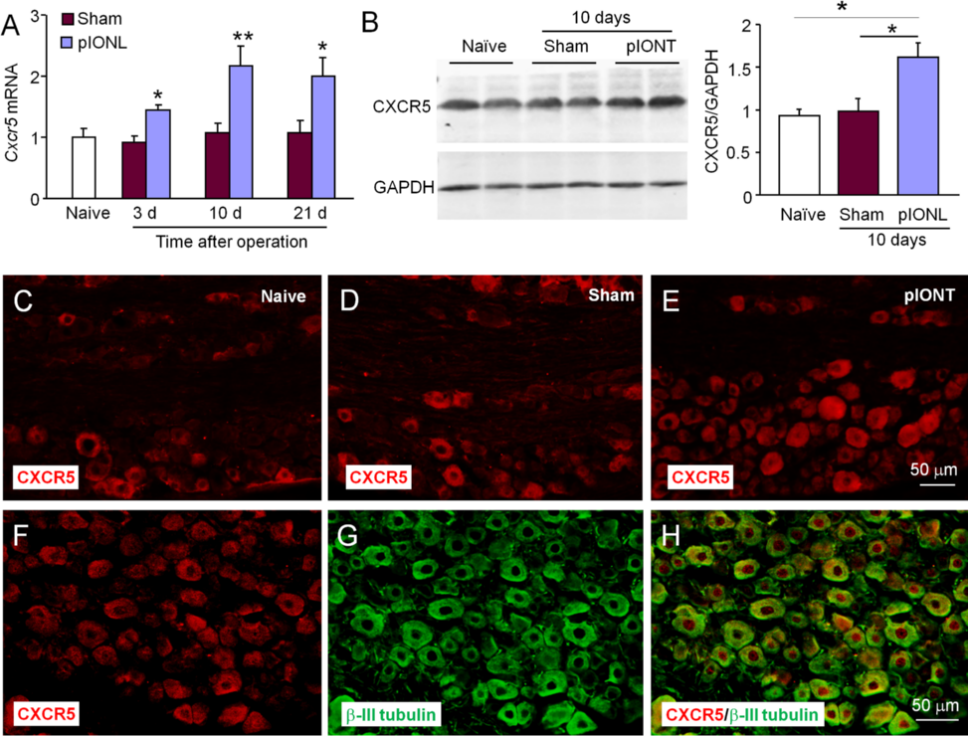
pIONL induces persistent CXCR5 expression in the TG. **a** The time course of *Cxcr5* mRNA expression in the ipsilateral TG from naïve, sham, and pIONL-operated mice. pIONL increased *Cxcr5* expression at 3, 10, and 21 days, compared to sham. *P < 0.05, **P < 0.01. Two-way ANOVA followed by Bonferroni test. **b** Western blot shows increased CXCR5 protein level 10 days after pIONL, compared to sham. *P < 0.05. One-way ANOVA followed by Bonferroni test. **c** Representative images of CXCR5 immunofluorescence in the TG. CXCR5-IR was low in naïve mice (**c**) and sham mice (**d**), but increased in the TG of pIONL mice (**e**). **f**–**h** Double staining of CXCR5 (**f**) and neuronal marker β-III tubulin (**g**) shows the neuronal expression of CXCR5

CXCR5 was reported to be the sole receptor for CXCL13 [25]. We then checked the time course of *Cxcr5* mRNA expression in the TG. pIONL induced persistent *Cxcr5* mRNA upregulation, which started at day 3 and maintained at day 21 (*P* < 0.05 or 0.01, pIONL vs. sham, two-way ANOVA followed by Bonferroni test, Fig. 4a). Western blot showed that CXCR5 protein level was significantly increased in pIONL mice at 10 days (*P* < 0.05, pIONL vs. sham or naive, one-way ANOVA followed by Bonferroni test, Fig. 4b). Immunostaining further revealed that CXCR5 had low expression in the TG in naïve mice (Fig. 4c) and sham-operated mice (Fig. 4d). CXCR5-IR was markedly increased 10 days after pIONL (Fig. 4e). In addition, CXCR5 was highly colocalized with β-III tubulin (Fig. 4f–h), indicating the neuronal expression of CXCR5 in the TG.

### Deletion of CXCR5 or inhibition of CXCR5 by shRNA lentivirus reduces pIONL-induced mechanical allodynia

**Fig. 5:**
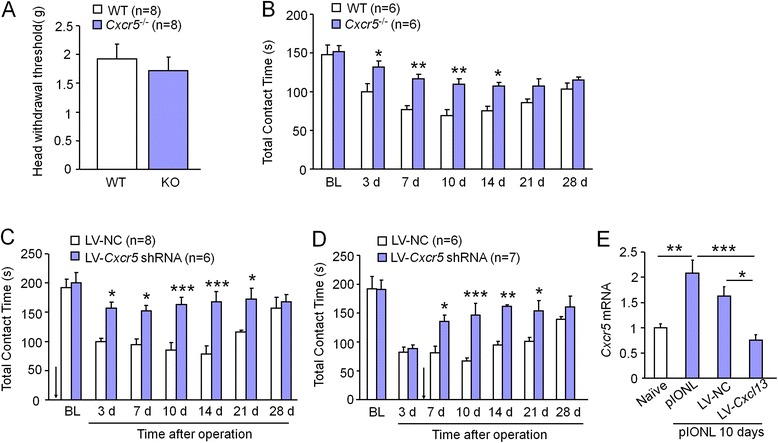
CXCR5 is essential for pIONL-induced mechanical allodynia. **a** Mechanical sensitivity assessed by Von Frey test was indistinguishable in WT and KO mice. **b** pIONL-induced mechanical allodynia was significantly reduced in *Cxcr5* KO mice, compared to WT mice. *P < 0.05, **P < 0.01. Two-way repeated measures ANOVA followed by Bonferroni test. **c** Pretreatment with LV-*Cxcr5* shRNA increased the contact time between 3 and 21 days after the operation, compared to LV-NC-injected mice. *P < 0.05, ***P < 0.001. Two-way repeated measures ANOVA followed by Bonferroni test. **d** Posttreatment with LV-*Cxcr5* shRNA increased the contact time between 7 and 21 days after the operation, compared to LV-NC treatment. *P < 0.05, **P < 0.01, ***P < 0.001. Two-way repeated measures ANOVA followed by Bonferroni test. **e** Real-time PCR assay of *Cxcr5* shows that pretreatment with LV-*Cxcr5* shRNA inhibited pIONL-induced *Cxcr5* upregulation. *P < 0.05, **P < 0.01, ***P < 0.001. Student’s *t* test

To determine the role of CXCR5 in trigeminal neuropathic pain, we checked pain behaviors in wild-type (WT; C57Bl/6) mice and *Cxcr5*^−/−^ (KO) mice. Acute mechanical sensitivity, as evaluated by Von Frey filament (Fig. 5a) was comparable between the two genotypes (*P* > 0.05, Student’s *t* test). We then tested mechanical allodynia by orofacial operant behavioral assessment after pIONL. pIONL-induced mechanical allodynia was significantly reduced at days 3, 7, 10, and 14 in *Cxcr5* KO mice compared to WT mice (*P* < 0.001, two-way repeated measures ANOVA, Fig. 5b)

To further check the role of CXCR5 in maxillary division of TG in the pathogenesis of neuropathic pain, we injected *Cxcr5* shRNA lentivirus (LV-*Cxcr5* shRNA and LV-NC) into the TG 10 days before or 3 days after pIONL in ICR mice. Pretreatment with LV-*Cxcr5* shRNA blocked pIONL-induced mechanical allodynia from 3 to 21 days after pIONL (*P* < 0.001, two-way repeated measures ANOVA, Fig.5c). Posttreatment with LV-*Cxcr5* shRNA alleviated mechanical allodynia from day 7 to day 21 (*P* < 0.001, two-way repeated measures ANOVA, Fig. 5d). qPCR further confirmed the knockdown effect of LV-*Cxcr5* shRNA on the expression of *Cxcr5* mRNA (*P* < 0.05, LV-*Cxcr5* shRNA vs. LV-NC, Fig. 5e).

### pIONL induces ERK activation in the TG of WT mice

**Fig. 6:**
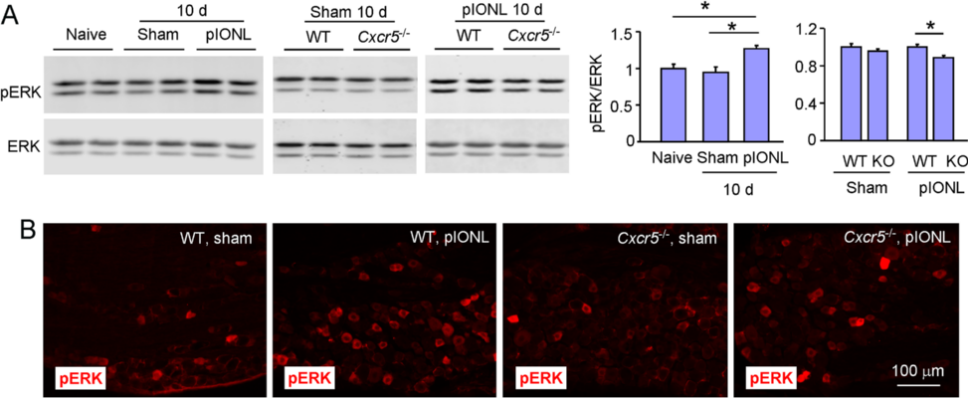
pIONL induces the activation of ERK in the TG. **a** Western blot shows the expression of pERK in naive, sham, and pIONL mice. pIONL increased the expression of pERK expression in WT mice, and the increase was reduced in *Cxcr5* KO mice. *P < 0.05. One-way ANOVA followed by Bonferroni test (*left histogram*) or Student’s *t* test (right histogram). **b** Representative images of pERK immunofluorescence in the TG of WT and KO mice 10 days after the operation. pERK-IR was increased after pIONL in WT mice, but not in KO mice

Our recent study showed that ERK is an important downstream of CXCL13/CXCR5 signaling in the spinal cord [11]. We then checked the activation of ERK in the TG after pIONL. Western blot showed that, compared to naive or sham-operated animals, pIONL increased pERK expression in the TG 10 days after pIONL (*P* < 0.05, vs. naïve or sham, one-way ANOVA followed by Bonferroni test, Fig. 6a). In addition, pERK expression was comparable between WT and *Cxcr5* KO mice after sham operation, but pERK was significantly reduced in KO mice after pIONL (*P* < 0.05, vs. WT, Student’s *t* test). Immunostaining showed that pERK had low expression in WT mice after sham operation, but was increased in WT mice after pIONL. However, similar expression of pERK was observed in both sham-operated and pIONL KO mice (Fig. 6b).

### pIONL-induced mechanical allodynia is mediated by ERK-dependent proinflammatory cytokines production in the TG

**Fig. 7:**
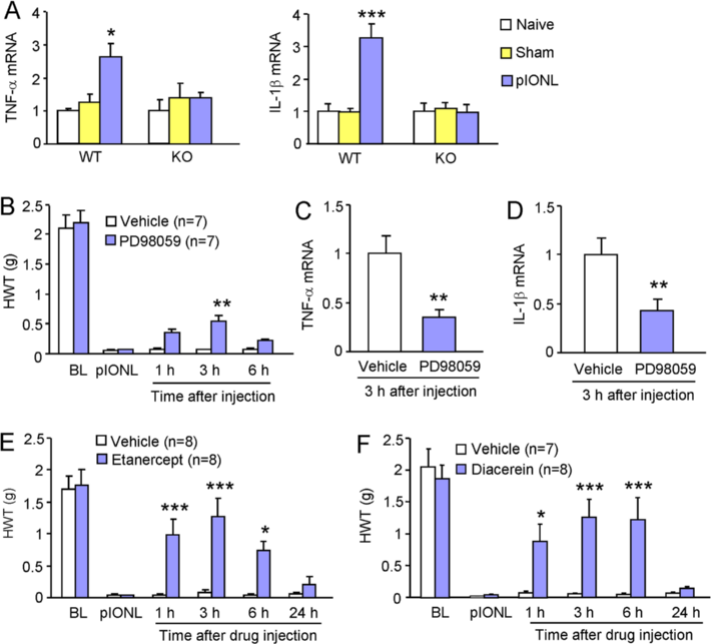
pIONL-induced mechanical allodynia is mediated by ERK-dependent proinflammatory cytokines production in the TG. **a** pIONL increased TNF-α and IL-1β expression 10 days after pIONL in WT mice, but not in *Cxcr5* KO mice. *P < 0.05, ***P < 0.001. One-way ANOVA followed by Bonferroni test. **b** Intra-TG injection of MEK inhibitor, PD98059 10 days after pIONL attenuated pIONL-induced mechanical allodynia at 3 h. **P < 0.01. Two-way repeated measures ANOVA followed by Bonferroni test. The same treatment reduced the expression of TNF-α (**c**) and IL-1β (**d**). **P < 0.01, Student’s *t* test. **e** Intra-TG injection of TNF-α inhibitor, Etanercept 10 days after pIONL alleviated pIONL-induced mechanical allodynia. The effect was shown 1 h after injection and maintained for more than 6 h. *P < 0.05, ***P < 0.001. Two-way repeated measures ANOVA followed by Bonferroni test. **f** Intra-TG injection of IL-1β inhibitor, Diacerein 10 days after pIONL alleviated pIONL-induced mechanical allodynia from 1 to 6 h. *P < 0.05, ***P < 0.001. Two-way repeated measures ANOVA followed by Bonferroni test

We further checked the downstream of ERK underlying pIONL-induced orofacial mechanical allodynia. TNF-α and IL-1β are important proinflammatory cytokines in regulating chronic pain in both peripheral nervous system and central nervous system [26]. We checked TNF-α and IL-1β mRNA expression in the TG 10 days after pIONL or sham operation. As shown in Fig. 7a, pIONL increased TNF-α and IL-1β mRNA expression in the TG of WT mice (TNF-α, *P* < 0.05; IL-1β, *P* < 0.001, pIONL vs. sham), but not in KO mice. These data suggest that pIONL induces CXCR5-dependent TNF-α and IL-1β expression.

To assess whether the production of TNF-α and IL-1β is dependent on ERK pathway, we injected MEK inhibitor PD98059 into the TG 10 days after pIONL. PD98059 (10 μg) [27] attenuated pIONL-induced mechanical allodynia 3 h after injection (*P* < 0.01, two-way repeated measures ANOVA, Fig. 7b) and reduced TNF-α and IL-1β mRNA expression (*P* < 0.01, PD98059 vs. vehicle, Fig. 7c, d), indicating that TNF-α and IL-1β are the downstream of ERK pathway in the TG.

We then checked the analgesic effect of the TNF-α inhibitor (Etanercept) and IL-1β inhibitor (Diacerein) on pIONL-induced mechanical allodynia. Intra-TG injection of Etanercept (1 μg) [21] 10 days after pIONL significantly alleviated pIONL-induced mechanical allodynia, with the effect maintained for more than 6 h (*P* < 0.001, two-way repeated measures ANOVA, Fig. 7e). Similarly, intra-TG injection of Diacerein (5 μg) [28] also attenuated pIONL-induced mechanical allodynia at 1, 3, and 6 h (*P* < 0.001, two-way repeated measures ANOVA, Fig. 7f). These results suggest that proinflammatory cytokines TNF-α and IL-1β are important in mediating orofacial neuropathic pain.

### Intra-TG injection of CXCL13 induces CXCR5/ERK-dependent mechanical allodynia and proinflammatory cytokines production

**Fig. 8:**
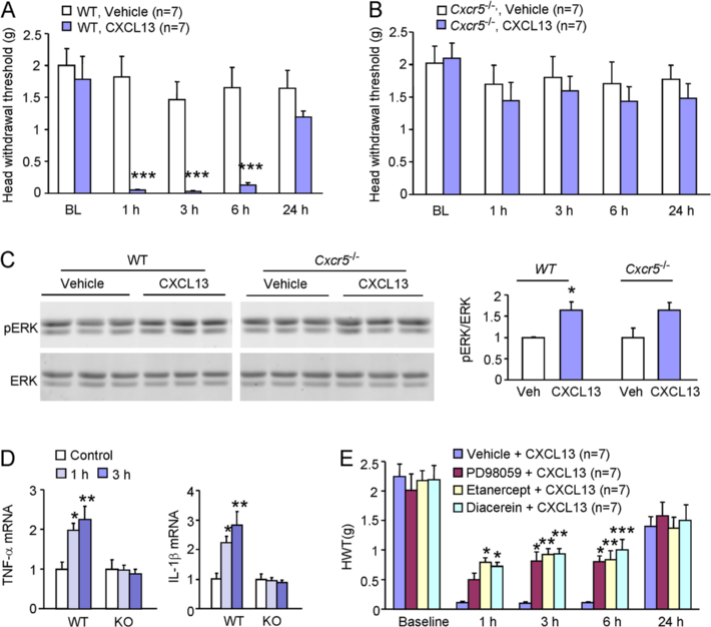
Intra-TG injection of CXCL13 induces CXCR5/ERK-dependent mechanical allodynia and proinflammatory cytokines production. **a**, **b** Intra-TG injection of CXCL13 (100 ng) induced mechanical allodynia in WT mice (**a**), but not in *Cxcr5* KO mice (**b**). ***P < 0.001, WT-vehicle vs. WT-CXL13. Two-way repeated measures ANOVA followed by Bonferroni test. **c** Western blot showing that intra-TG CXCL13 significantly increased pERK expression in the TG in WT mice, but not in *Cxcr5* KO mice. *P < 0.05. Student’s *t* test. **d** Intra-TG injection of CXCL13 increases TNF-α and IL-1β expression at 1 and 3 h in WT mice, but not in *Cxcr5* KO mice. *P < 0.05, **P < 0.01. One-way ANOVA followed by Bonferroni test. **e** Intra-TG injection of PD98059, Etanercept, or Diacerein partially blocked intra-TG CXCL13-induced mechanical allodynia. *P < 0.05, **P < 0.01, ***P < 0.001. Two-way repeated measures ANOVA followed by Bonferroni test

To investigate whether CXCL13 is sufficient to induce orofacial pain, we injected CXCL13 into the TG in WT and *Cxcr5* KO mice. As shown in Fig. 8a, CXCL13 (100 ng), but not vehicle (PBS)-induced mechanical allodynia in WT mice for more than 6 h (*P* < 0.001, two-way repeated measures ANOVA), whereas *Cxcr5* KO mice failed to develop the mechanical allodynia by CXCL13 (Fig. 8b).

We further checked the expression of pERK in the TG after CXCL13 injection. Western blot showed that CXCL13 induced significant pERK expression in the TG 1 h after injection in WT mice (*P* < 0.05, Student’s *t* test), but not in *Cxcr5* KO mice (*P* > 0.05, Student’s *t* test, Fig. 8c). These data suggest that ERK is the downstream of CXCL13/CXCR5 signaling in the TG.

To examine whether CXCL13 could increase TNF-α and IL-1β mRNA expression in the TG, we checked the mRNA level of TNF-α and IL-1β in the TG 1 and 3 h after CXCL13 injection. It showed that the expression of TNF-α and IL-1β mRNA was significantly increased at both 1 and 3 h in the WT mice (*P* < 0.01, one-way ANOVA, Fig. 8d). However, the mRNA level of TNF-α and IL-1β was not significantly changed in *Cxcr5* KO mice (*P* > 0.05, one-way ANOVA, Fig. 8d), suggesting that CXCL13-induced TNF-α and IL-1β expression is dependent on CXCR5.

To check if CXCL13-induced mechanical allodynia is dependent on ERK activation and TNF-α and IL-1β production, PD98059, Etanercept, or Diacerein was injected into the TG and followed by CXCL13 injection 1 h later. Behavioral test showed that pretreatment with these drugs partially attenuated CXCL13-induced mechanical allodynia (*P* < 0.001, two-way repeated measures ANOVA, Fig. 8e). In addition, PD98059 pretreatment also reduced the mRNA level of TNF-α (1 ± 0.27 vs. 0.27 ± 0.09, vehicle + CXCL13 vs. PD98059 + CXCL13, *P* < 0.05, Student’s *t* test) and IL-1β (1 ± 0.23 vs. 0.31 ± 0.06, vehicle + CXCL13 vs. PD98059 + CXCL13, *P* < 0.05, Student’s *t* test), which was examined 3 h after CXCL13 injection. These data indicate that CXCL13-induced mechanical allodynia is dependent on CXCR5/ERK/TNFα and IL-1β pathway.

## Discussion

This is the first study that examines in detail the role of CXCL13/CXCR5 signaling in the maxillary part of the TG in pIONL-induced mechanical allodynia. We have made the following new findings. First, pIONL persistently increased CXCL13 and CXCR5 expression in TG neurons. Consistently, inhibition of CXCL13/CXCR5 before or after pIONL attenuated pIONL-induced mechanical allodynia. Second, pIONL induced ERK activation and ERK-dependent TNF-α and IL-1β production in the TG of WT mice. TNF inhibitor and IL-1 inhibitor alleviated pIONL-induced mechanical allodynia. Finally, intra-TG injection of CXCL13 induced mechanical allodynia, ERK activation, and TNF-α and IL-1β production. Inhibition of ERK signaling or TNF-α or IL-1β attenuated CXCL13-induced mechanical allodynia. Taken together, our results indicate that CXCL13/CXCR5 signaling in the TG is involved in orofacial neuropathic pain, at least partially, via ERK-mediated proinflammatory cytokines production.

### CXCL13 upregulation in the TG contributes to neuropathic pain

Chemokines, which comprise a family of >50 family members, have been recognized for its pivotal role in the pathogenesis of neuropathic pain [2, 29, 30]. Several chemokines including CCL2, CCL7, and CXCL1 are upregulated in the spinal cord following nerve injury and contribute to neuropathic pain [21, 31, 32]. By using mouse gene expression microarrays, we recently found that 10 chemokines (including CCL2, CCL7, CXCL1, and CXCL13) whose expression was increased more than three fold in the spinal cord after SNL. Among them, CXCL13 is the most upregulated gene with 47-fold increase [11]. In the present study, we for the first time found that CXCL13 mRNA in the TG was upregulated 3 days after ligation and maintained for more than 21 days. Western blot and immunostaining further showed the upregulated CXCL13 protein in the ipsilateral TG. In agreement with our results, Strong et al. reported that CXCL13 mRNA was increased in the DRG 14 days after local inflammation of DRG or 3 days after SNL or modified SNL model in rats [12]. Previous studies have shown that CXCL13 was induced in some microglia, macrophages, and endothelial cells in the CNS after infection [33, 34] or in infiltrating dendritic cells in EAE mice [8, 9]. Our recent data showed that CXCL13 was predominantly produced by spinal neurons after SNL [14]. Here, we also found that CXCL13 was expressed in TG neurons, indicating the neuronal expression of CXCL13 in both TG and spinal cord.

By using shRNA lentivirus injection to specific knockdown the expression of CXCL13 in the maxillary part of the TG, we found that both pretreatment and posttreatment with CXCL13 shRNA lentivirus effectively attenuated pIONL-induced mechanical allodynia. These data suggest that CXCL13 is necessary for the development and maintenance of orofacial neuropathic pain.

### CXCR5 upregulation in the TG contributes to neuropathic pain

It has been demonstrated that chemokines are involved in chronic pain modulation through neuronal-glial interactions [5]. For example, chemokines CX3CL1 and CCL21 are expressed in primary sensory neurons and induce microglial activation via their microglial receptors CX3CR1 and CCR7/CXCR3, respectively [35–38]. Our previous studies have demonstrated astroglial-neuronal interaction in neuropathic pain via respective expression of CCL2 and CXCL1 in spinal astrocytes and CCR2 and CXCR2 in spinal neurons [21, 31]. We recently reported that CXCR5, the receptor of CXCL13, was expressed in spinal astrocytes and mediated a novel form of neuronal-glial interaction in neuropathic pain [11]. In this study, we found that CXCR5 was persistently (>21 days) increased after pIONL with the expression in TG neurons, suggesting that CXCL13 and CXCR5 in the TG mediate orofacial neuropathic pain through distinct mechanism.

Our behavioral data showed that pIONL-induced mechanical allodynia was reduced from 3 to 14 days in *Cxcr5*-deficient mice. In addition, using shRNA lentivirus injection to specific knockdown the expression of CXCR5 in the maxillary part of the TG, we found that both pretreatment and posttreatment with CXCR5 shRNA lentivirus effectively attenuated pIONL-induced mechanical allodynia. These data suggest that CXCL13/CXCR5 signaling is necessary for the development and maintenance of orofacial neuropathic pain. Moreover, intra-TG injection of CXCL13 induced CXCR5-dependent mechanical allodynia for more than 6 h, indicating that activation of CXCR5 in the TG is also sufficient to induce orofacial pain hypersensitivity.

### CXCR5-ERK cascade contributes to trigeminal neuropathic pain

CXCR5 belongs to G protein-coupled receptors (GPCR) family, and activation of GPCR can regulate many signaling pathways. MAPKs are key components in GPCR-induced intracellular signaling [39] and have been implicated in mediating chronic pain in both DRG and spinal cord [15]. Peripheral inflammation by complete Freund’s adjuvant (CFA) [40] or nerve injury by SNL [41–43] induces ERK activation in DRG neurons. In addition, the temporomandibular joint inflammation increases pERK expression in the TG [44]. Here, we found that pIONL increased pERK expression in TG neurons of WT mice. However, the expression of pERK was reduced in *Cxcr5*-deficient mice. Moreover, intra-TG injection of CXCL13 induced CXCR5-dependent pERK upregulation in the TG. Our recent study showed that CXCL13 activated ERK in spinal astrocytes through CXCR5 [11]. These data suggest the important role of ERK in mediating CXCL13/CXCR5 signaling in both TG and spinal cord.

Studies have shown that intrathecal injection of MEK inhibitor alleviated CFA-induced inflammatory pain and peripheral nerve injury-induced neuropathic pain [45, 46]. We showed that intra-TG injection of PD98059 attenuated pIONL- or CXCL13-induced mechanical allodynia. It was known that ERK mediates the expression of inflammatory mediators, including growth factors [45] and proinflammatory cytokines (e.g., IL-1β) [47]. We also found that pIONL increased TNF-α and IL-1β expression in the TG of WT mice, but the expressions were reduced in *Cxcr5* KO mice. In addition, intra-TG injection of CXCL13 induced CXCR5/ERK-dependent TNF-α and IL-1β upregulation. TNF-α and IL-1β are important proinflammatory cytokines that mediate chronic pain in both DRG and spinal cord [48]. Behavioral data further showed that inhibition of TNF-α or IL-1β alleviated pIONL- or CXCL13-induced pain hypersensitivity, indicating the important role of proinflammatory cytokines in mediating orofacial neuropathic pain.

## Conclusions

In this study, we provided the first evidence that CXCL13 and CXCR5 were involved in pIONL-induced orofacial mechanical allodynia via ERK-mediated proinflammatory cytokines production in the TG. Thus, targeting the CXCL13/CXCR5/ERK/TNF-α and IL-1β pathway in the TG may provide a novel therapeutic approach for the treatment of the trigeminal neuralgia.

## Abbreviations

CXCL13, chemokine C-X-C motif ligand 13; CXCR5, chemokine C-X-C motif receptor 5; CNS, central nervous system; DRG, dorsal root ganglion; EAE, experimental autoimmune encephalomyelitis; ERK, extracellular signal-regulated kinase; GAPDH, glyceraldehyde-3-phosphate dehydrogenase; GFP, green fluorescent protein; HWT, head-withdrawal threshold; JNK, c-Jun *N*-terminal kinase; IL-1β, interleukin-1β; MAPK, mitogen-activated protein kinase; ION, infraorbital nerve; PBS, phosphate-buffered saline; pIONL, partial infraorbital nerve ligation; TNF-α, tumor necrosis factor-α; TG, trigeminal ganglion
